# Supplementary material for: Treadmill Exercise Reduces Neuroinflammation, Glial Cell Activation and Improves Synaptic Transmission in the Prefrontal Cortex in 3 × Tg-AD Mice
Source: Int J Mol Sci. 2022 Oct 21;23(20):12655. doi: 10.3390/ijms232012655 (PMC9604030; doi:10.3390/ijms232012655)
Supplement: Supplementary file 1 [file ijms-23-12655-s001.zip › ijms-1975356-supplementary.pdf]

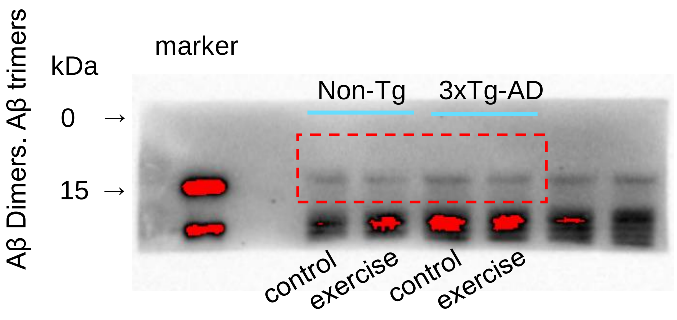

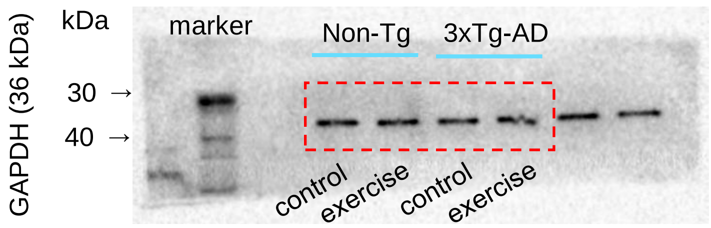

Supplementary Figure S1. Full-length Western blots of the Aβ Dimers and Aβ Trimers expression data shown in Figure 1. Red boxes indicate the bands that were cropped for the representative images shown in Figure 1.

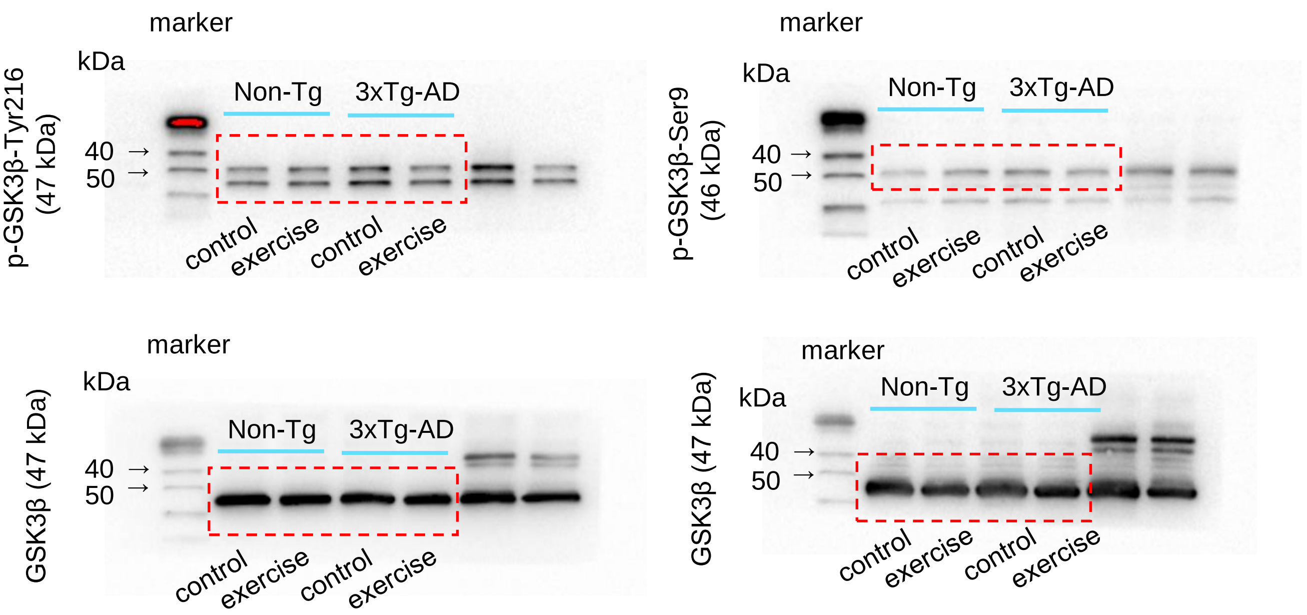

Supplementary Figure S2. Full-length Western blots of the GSK3β, p-GSK3β-Tyr216 and p-GSK3β-Ser9 expression data shown in Figure 2. Red boxes indicate the bands that were cropped for the representative images shown in Figure 2.

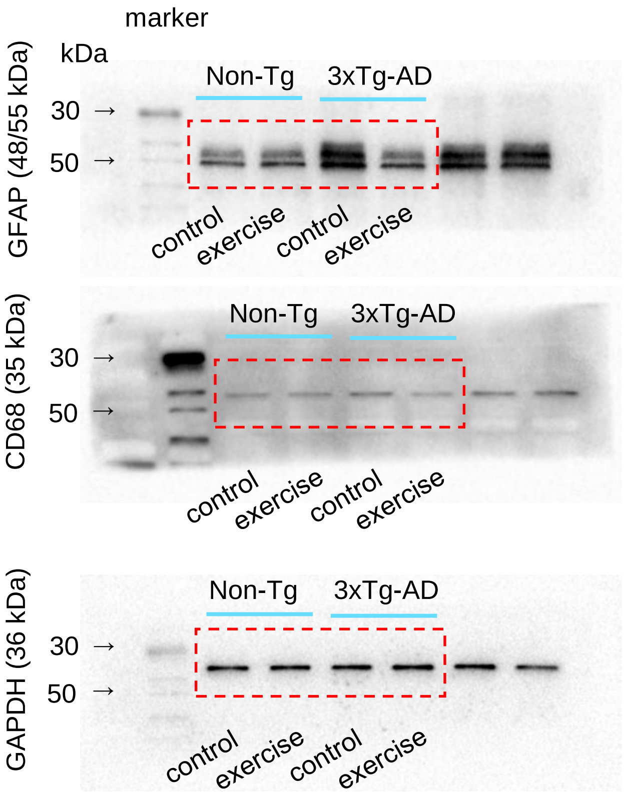

Supplementary Figure S3. Full-length Western blots of the GFAP and CD68 expression data shown in Figure 3. Red boxes indicate the bands that were cropped for the representative images shown in Figure 3.

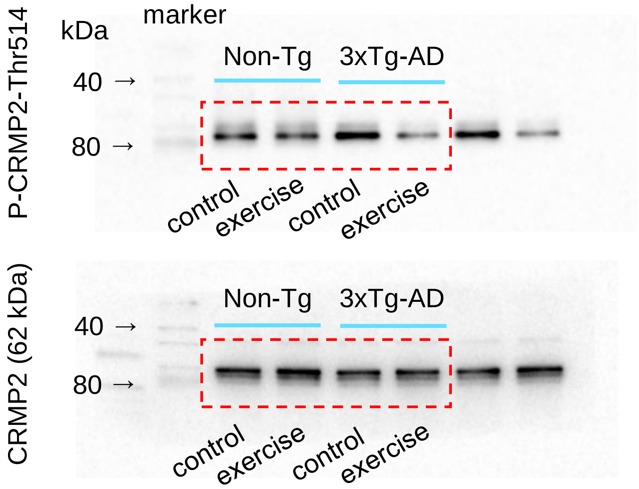

Supplementary Figure S4. Full-length Western blots of the CRMP2 and p-CRMP2-Thr514 expression data shown in Figure 4. Red boxes indicate the bands that were cropped for the representative images shown in Figure 4.
